# The Activation of Aldehyde Dehydrogenase 2 (ALDH2) by Alda-1 and Flurbiprofen as a Common Mechanism to Reduce Alcohol Intake in Rats

**DOI:** 10.3390/ijms27073248

**Published:** 2026-04-03

**Authors:** Juan Manuel Torres, Carolina Ponce, Vicente Pérez, Ignacio Gutiérrez-Vega, María Elena Quintanilla, David Vásquez, Mario Rivera-Meza

**Affiliations:** 1 Department of Pharmacological and Toxicological Chemistry, Faculty of Chemical Sciences and Pharmacy, University of Chile, Santiago 8380494, Chile; juanmanut35@hotmail.com (J.M.T.); c.poncehenriquez24@gmail.com (C.P.); vicente.perez.p@ug.uchile.cl (V.P.); ignacio.gutierrez.v@ug.uchile.cl (I.G.-V.); dvasquez@ciq.uchile.cl (D.V.); 2 Program of Molecular and Clinical Pharmacology, Faculty of Medicine, University of Chile, Santiago 8380494, Chile; equintanilla@uchile.cl; 3 Specialized Center for Prevention of Substance Use and Treatment of Addictions (CESA), Faculty of Medicine, University of Chile, Santiago 8380494, Chile

**Keywords:** alcohol, flurbiprofen, Alda-1, ALDH2, ibuprofen, PC-12 cells, UChB rats, molecular docking

## Abstract

Excessive alcohol consumption causes millions of deaths annually, yet current pharmacological treatments for alcohol use disorders show limited efficacy and poor adherence, creating an urgent need for new therapeutic alternatives. Aldehyde dehydrogenase 2 (ALDH2) metabolizes acetaldehyde, a key mediator of the rewarding effects of alcohol in the brain, making ALDH2 activation a promising therapeutic target. This study investigated whether flurbiprofen, an FDA-approved nonsteroidal anti-inflammatory drug that activates ALDH2, reduces alcohol intake compared to the experimental ALDH2 activator Alda-1 and the structurally similar NSAID ibuprofen. Male alcohol-preferring UChB rats received oral flurbiprofen (2.5–10 mg/kg), Alda-1 (5 mg/kg), or ibuprofen (5 mg/kg) during acquisition and chronic phases of voluntary alcohol consumption under a two-bottle free-choice paradigm. Both flurbiprofen and Alda-1 reduced alcohol intake by approximately 60% and similarly increased ALDH2 activity 3–4-fold in brain and liver tissues. Ibuprofen showed modest effects (25% alcohol intake reduction). In vitro assays confirmed that flurbiprofen and Alda-1, but not ibuprofen, activated ALDH2 in PC-12 cells. Enzymatic assays and molecular docking revealed that Alda-1 lacks cyclooxygenase-inhibitory activity, unlike flurbiprofen, suggesting that ALDH2 activation is the primary mechanism underlying reduced alcohol consumption. These findings identify flurbiprofen as a clinically available ALDH2 activator with significant translational potential for treating alcohol use disorders.

## 1. Introduction

Excessive alcohol consumption causes 3 million deaths annually, with young people aged 20 to 39 accounting for 13.5% of this number [1]. This problem generates a profound socioeconomic impact and disproportionately affects vulnerable populations, who record the highest rates of alcohol-related deaths and hospitalizations [1]. The current treatment of alcohol use disorders combines psychotherapeutic, psychosocial, and/or pharmacological interventions. However, the available pharmacological options, naltrexone, acamprosate, and disulfiram, present low adherence, limited efficacy, and significant adverse effects, which frequently lead to treatment abandonment [2,3]. Therefore, the development of new, more effective therapeutic alternatives that offer better clinical outcomes constitutes an urgent health need.

In humans, approximately 90% of ingested alcohol is metabolized by the liver. Alcohol (ethanol) is initially converted to acetaldehyde mainly via enzymatic pathways [4]. The primary enzymatic system responsible for alcohol oxidation to acetaldehyde is alcohol dehydrogenase (ADH), with cytochrome P450-dependent alcohol oxidation occurring to a lesser extent. Subsequently, acetaldehyde is converted into acetate through the action of mitochondrial aldehyde dehydrogenase (ALDH2). This enzyme is present in all tissues but is found at higher concentrations in the liver. It should be noted that the blood–brain barrier (BBB) restricts the entry of acetaldehyde into the brain [5] due to the presence of ALDH2 in the cerebral microvasculature [6]. Consequently, the concentration of acetaldehyde in the central nervous system (CNS) depends mainly on its local production from alcohol. The oxidation of alcohol to acetaldehyde in the brain is mediated 60% by catalase and, to a lesser extent, by CYP2E1; in contrast, ADH expression in the CNS is minimal [7].

A characteristic of addictive substances, including alcohol, lies in their ability to stimulate the CNS. Various investigations have shown that, despite its peripheral harmful effects, acetaldehyde produced in the brain exerts a significant influence, with a marked impact on reinforcement [8]. Studies by Rodd et al. [9] demonstrated that rats self-administer acetaldehyde in the brain at micromolar levels, confirming its positive effects on motivation and suggesting that acetaldehyde may be implicated in the motivational actions of alcohol [10,11]. Indeed, pharmacogenetic studies have shown that reducing brain acetaldehyde generation by silencing catalase expression, or accelerating its elimination from the brain by overexpressing ALDH2, is associated with a marked reduction in voluntary alcohol intake in rats [12,13].

A novel class of organic compounds has been identified that enhances ALDH2 catalytic activity by stabilizing the interaction between the cofactor and substrate at the active site of the enzyme [14]. Alda-1, a prototype enzymatic activator from this class, has demonstrated protective effects against multiple pathological conditions in animal models, including myocardial infarction, stroke, and acute lung injury. These protective effects stem from the ability of Alda-1 to enhance ALDH2-mediated clearance of toxic aldehydes, thereby reducing cell death, inflammation, and tissue damage across multiple organ systems [15,16,17].

Our laboratory has shown that administration of Alda-1 to rats significantly increases ALDH2 activity in both hepatic and brain tissues [18]. Furthermore, Alda-1 markedly reduces alcohol intake in animals following chronic exposure (>90 days) to alcohol [18] and suppresses relapse-like drinking behavior in rats subjected to alcohol deprivation after chronic exposure [19]. This reduction in consumption would be associated with lower brain acetaldehyde levels, attributable to increased ALDH2 activity.

Hosoi et al. [20] reported that flurbiprofen, a nonsteroidal anti-inflammatory drug (NSAID) derived from propionic acid, increases ALDH2 catalytic velocity without altering enzyme expression or stability—effects comparable to those of Alda-1. Given that Alda-1 is an experimental compound, flurbiprofen offers a significant translational advantage for exploring its potential protective effects against alcohol consumption, as it is an FDA-approved medication already in clinical use. Although previous studies have evaluated the interaction between NSAIDs and alcohol at the metabolism and toxicology level [21,22,23], there are no reports about the effects of flurbiprofen or other NSAIDs on alcohol intake behavior in animal models. Therefore, the present study aims to determine whether flurbiprofen, compared to Alda-1 and ibuprofen, affects alcohol consumption behavior in the alcohol-preferring UChB rat model. Additionally, it explores the molecular mechanisms through which these drugs exert their effects.

## 2. Results

**Figure 1:**
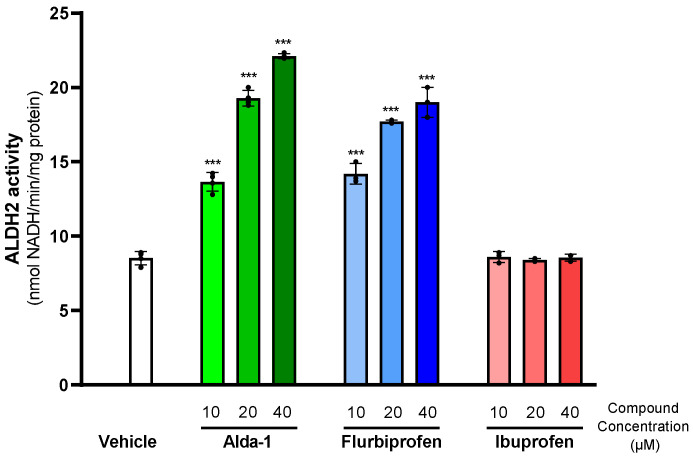
**ALDH2 activity in PC-12 cells incubated with different concentrations of Alda-1, flurbiprofen, and ibuprofen.** Cultures of PC-12 cells were incubated for 2 h with 10, 20, or 40 μM of Alda-1 (green bars), flurbiprofen (blue bars), ibuprofen (red bars), or vehicle (0.5% DMSO, white bar). ALDH2 activity was expressed as nmol NADH/min/mg protein ± SEM of three independent experiments performed in duplicate. A one-way ANOVA analysis revealed significant differences for Alda-1 and flurbiprofen treatments (*p* < 0.001. *** *p* < 0.001 vs. vehicle, Dunnett’s test).

The effects of Alda-1, flurbiprofen, and ibuprofen on ALDH2 activity in vitro were assessed by exposing PC12 cell cultures to these compounds at concentrations of 10, 20, and 40 μM (Figure 1). Results showed that both Alda-1 and flurbiprofen increased ALDH2 enzymatic activity in PC12 cells in a dose-dependent manner compared with vehicle (One-way ANOVA; *F*_(3, 12)_ = 644.8, *p* < 0.001 for Alda-1; *F*_(3, 9)_ = 194.9, *p* < 0.001 for flurbiprofen). For Alda-1 at 40 μM, ALDH2 activity reaches 22.1 ± 0.2 nmol NADH/min/mg protein, a 2.6-fold increase compared to vehicle-treated cells (Dunnett’s test, *q* = 40.4, *p* < 0.001). Similarly, a concentration of 40 μM flurbiprofen results in an ALDH2 activity of 19.0 ± 1.0 nmol NADH/min/mg protein, a 2.2-fold increase compared with vehicle-treated cells (Dunnett’s test, *q* = 21.7, *p* < 0.001). In the case of ibuprofen, no increases in ALDH2 activity were observed at any of the studied concentrations (One-way ANOVA, *F*_(3, 9)_ = 0.2, *p* > 0.05).

**Figure 2:**
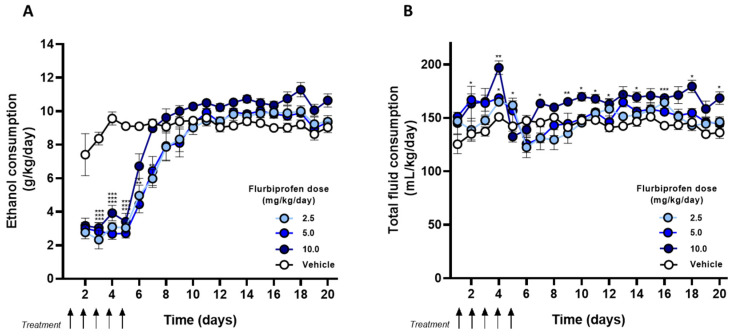
**Effect of different doses of flurbiprofen on the acquisition of alcohol intake in UChB rats.** Ethanol-naïve UChB male rats were treated daily (i.g.) for 5 days (black arrows) with flurbiprofen 2.5 mg/kg (*n* = 5; light blue circles), 5 mg/kg (*n* = 5; blue circles), 10 mg/kg (*n* = 5; dark blue circles) or vehicle (2% Arabic gum, 1.5 mL, *n* = 5; white circles). On day 2 of flurbiprofen administration, animals were given a 24 h free choice between 10% ethanol and water for 20 days. (**A**) Circles represent means ± SEM of daily ethanol intake (g/kg/day). A two-way RM ANOVA test indicates significant differences between groups during treatment (*p* < 0.001). (**B**) Circles represent means ± SEM of total fluid intake (mL/kg/day). A two-way RM ANOVA test indicates significant differences between groups during treatment (*p* < 0.05). (* *p* < 0.05, ** *p* < 0.01, *** *p* < 0.001 vs. vehicle, Bonferroni’s post hoc test).

Then, we studied the effect of daily oral administration of flurbiprofen at 2.5, 5, and 10 mg/kg (*n* = 5 animals/group) for 5 days on the acquisition of voluntary alcohol intake in UChB drinking rats. Results showed that all administered doses of flurbiprofen elicited a marked reduction of approximately 65% in alcohol intake compared to vehicle-treated animals (~3.0 vs. 8.5 g ethanol/kg/day), without a dose-dependent relationship in this protective effect (RM two-way ANOVA, *F*_(3, 16)_ = 66.5, *p* < 0.001) (Figure 2A). After discontinuing flurbiprofen administration, animals rapidly increase their alcohol consumption, reaching, after approximately 7 days, the level of consumption shown by the vehicle-treated group. Rats were then maintained under the two-bottle free-choice paradigm (water and 10% ethanol) until day 20. We also found that the administration of the different doses of flurbiprofen elicited a slight increase in total fluid intake compared to vehicle-treated animals during treatment (RM two-way ANOVA, *F*_(3, 16)_ = 6.3, *p* < 0.05) and after the discontinuation of drug administration (RM two-way ANOVA, *F*_(3, 16)_ = 13.3, *p* < 0.05) (Figure 2B).

**Figure 3:**
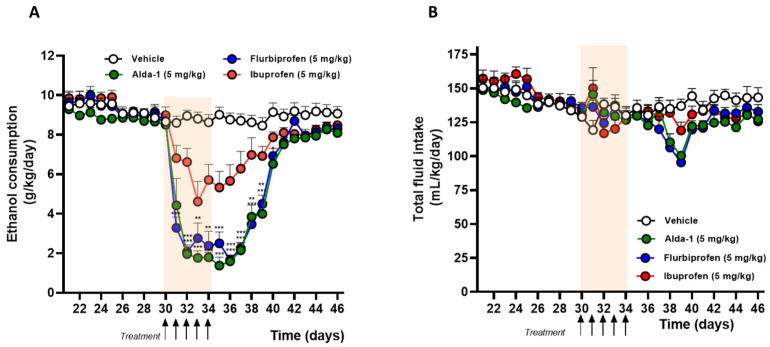
**Effect of flurbiprofen, Alda-1, and ibuprofen on the alcohol intake of UChB rats following chronic alcohol exposure.** UChB male rats were exposed to voluntary ethanol intake for 30 days. On day 30 animals were treated daily (i.g.) for 5 days (black arrows) with flurbiprofen 5 mg/kg (*n* = 5; blue circles), Alda-1 5 mg/kg (*n* = 5; green circles), ibuprofen 5 mg/kg (*n* = 5; red circles) or vehicle (2% Arabic gum, 1.5 mL, *n* = 5; white circles). After stopping drug administration, voluntary ethanol intake was monitored until day 46. (**A**). Circles represent means ± SEM of daily ethanol intake (g/kg/day). A two-way RM ANOVA indicates significant differences between groups during treatment (*p* < 0.001 for flurbiprofen vs. vehicle; *p* < 0.001 for Alda-1 vs. vehicle; *p* < 0.05 for ibuprofen vs. vehicle). (* p < 0.05, ** *p* < 0.01, *** *p* < 0.001 vs. vehicle, Bonferroni’s post hoc test) (**B**). Circles represent means ± SEM of total fluid intake (mL/kg/day). A two-way RM ANOVA test did not reveal significant differences between groups during treatment (*p* > 0.05).

On day 20 of the experiment, animals were randomly assigned to four new groups to further examine the effects of flurbiprofen, Alda-1, and ibuprofen on chronic alcohol intake. After 10 days of establishing baseline consumption (day 30), animals received daily oral doses of 5 mg/kg Alda-1, flurbiprofen, or ibuprofen, or an equivalent volume of vehicle (*n* = 5 per group), for 5 consecutive days (Figure 3A). The rats treated with flurbiprofen decreased their alcohol consumption by 58% compared to the vehicle group (3.7 ± 0.7 vs. 8.8 ± 0.1 g ethanol/kg/day; mean consumption days 30–40; RM two-way ANOVA, *F*_(1, 8)_ = 143.5, *p* < 0.001). Similarly, Alda-1 treatment was associated with a 61% reduction in alcohol intake compared with the vehicle group (3.4 ± 0.7 vs. 8.8 ± 0.1 g ethanol/kg/day; mean consumption days 30–40; RM two-way ANOVA, *F*_(1, 8)_ = 221.6, *p* < 0.001). In the case of ibuprofen, its administration was associated with a lower effect on alcohol intake, eliciting a reduction of 25% in comparison to the vehicle group (6.5 ± 0.4 vs. 8.8 ± 0.1 g ethanol/kg/day; mean consumption days 30–40; RM two-way ANOVA, *F*_(1, 8)_ = 10.3, *p* < 0.05). In all drug-treated groups, upon treatment discontinuation, animals slowly recover their alcohol intake, reaching levels comparable to those of the vehicle approximately 8 days after treatment cessation. We also found that the pharmacological treatment of the rats was not associated with changes in total fluid intake during the drug administration period (days 30–34) (RM two-way ANOVA, *F*_(3, 16)_ = 0.1, *p* > 0.05) nor throughout the entire period of chronic alcohol intake (days 22–46) (RM two-way ANOVA, *F*_(3, 16)_ = 0.8, *p* > 0.05) (Figure 3B).

**Figure 4:**
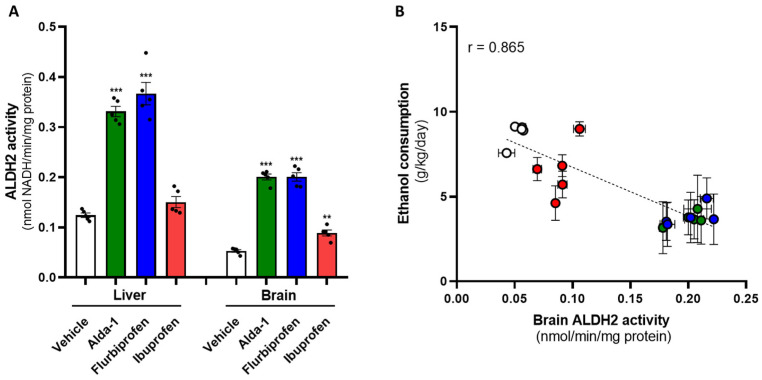
**Effect of the administration of flurbiprofen, Alda-1, and ibuprofen on the liver and brain ALDH2 activity of UChB rats.** Seven days after finishing alcohol drinking, animals were treated daily (i.g.) for 5 days with flurbiprofen 5 mg/kg (*n* = 5; blue symbols), Alda-1 5 mg/kg (*n* = 5; green symbols), ibuprofen 5 mg/kg (*n* = 5; red symbols) or vehicle (2% Arabic gum, 1.5 mL, *n* = 5; white symbols). Animals were then euthanized, and the brain and liver were excised for ALDH2 activity measurement. (**A**). Bars represent means ± SEM of ALDH2 activity (nmol NADH/min/mg protein) measured in duplicate for each animal. A one-way ANOVA indicates significant differences between treatments for each tissue analyzed (*p* < 0.001). (** *p* < 0.01, *** *p* < 0.001 vs. vehicle, Tukey’s test). (**B**). Circles represent the mean ± SEM of chronic alcohol consumption (g ethanol/kg/day) during the 5 days of treatment vs. the mean ± SEM of brain ALDH2 activity for each animal. Linear regression analysis of the slope (dotted line) indicates a significant inverse correlation with the factors (*p* < 0.001; not equal to 0).

Once the effect of the drugs on alcohol consumption in UChB rats was established, their impact on ALDH2 activity in the brain and liver was examined. To this end, after day 46, access to the alcohol bottle was removed, leaving only water available for 1 week. Then, animals were re-treated for 5 days with a daily dose of Alda-1 (5 mg/kg), flurbiprofen (5 mg/kg), ibuprofen (5 mg/kg), or an equivalent volume of vehicle. One hour after the last dose, the animals were euthanized, and the brain and liver were quickly collected for ALDH2 activity measurement. The results indicate that in both tissues, Alda-1 and flurbiprofen significantly increased ALDH2 activity compared to the vehicle group (one-way ANOVA; liver *F*_(3, 16)_ = 81.5, *p* < 0.001; brain *F*_(3, 16)_ = 157.8, *p* < 0.001) (Figure 4A). In the liver, administration of Alda-1 (5 mg/kg) and flurbiprofen (5 mg/kg) resulted in a 2.7- and 3-fold increase in ALDH2 activity compared with the vehicle group (Tukey’s test; Alda-1, *q* = 15.1, *p* < 0.001; flurbiprofen, *q* = 17.7, *p* < 0.001). The ALDH2 activity induced by Alda-1 and flurbiprofen showed no significant difference between them at the hepatic level (Tukey’s test, *q* = 2.6, *p* > 0.05). In the 5 mg/kg ibuprofen group, activity increased only 1.08-fold, which did not differ significantly from the vehicle group (Tukey’s test, *q* = 1.9, *p* > 0.05). In the brain, administration of Alda-1 and flurbiprofen resulted both in a 3.8-fold increase in ALDH2 activity compared with the vehicle group (Tukey’s test; Alda-1, *q* = 24.3, *p* < 0.001; flurbiprofen, *q* = 24.3, *p* < 0.001). The ALDH2 activity induced by Alda-1 and flurbiprofen did not differ significantly at the brain level (Tukey’s test, *q* = 0.03, *p* > 0.05). In the ibuprofen group, activity increased 1.7-fold compared with the vehicle group (Tukey’s test, *q* = 5.9, *p* < 0.01). After plotting the values of brain ALDH2 activity in treated animals against their corresponding voluntary alcohol intake over the 5 days of drug treatment, we found a significant negative correlation between both factors (r = −0.865, *p* < 0.001) (Figure 4B).

**Figure 5:**
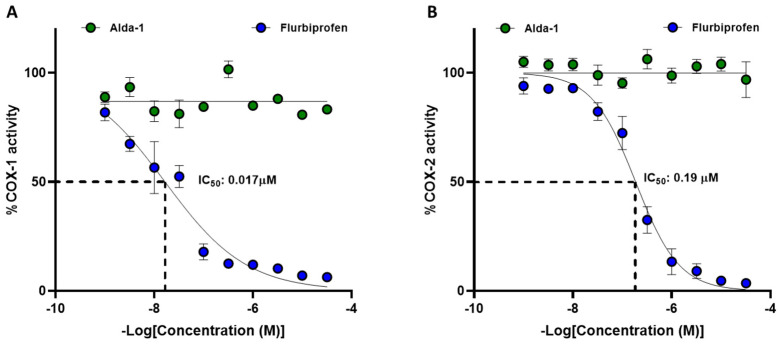
**Effect of different concentrations of Alda-1 and flurbiprofen on the enzymatic activity of COX-1 and COX-2.** Purified recombinant ovine COX-1 (**A**) or human COX-2 (**B**) were incubated in triplicate with Alda-1 (green circles) or flurbiprofen (blue circles) at concentrations of 0.001, 0.0032, 0.01, 0.032, 0.1, 0.32, 1, 3.2, 10, 32, and 100 μM. Circles represent the mean ± SEM of the remnant activity (%). Inhibitory concentrations 50 (IC_50_) for flurbiprofen on COX-1/2 were determined by nonlinear regression. Alda-1 did not show inhibitory actions on COX-1/2, and the corresponding IC_50_ could not be calculated. The dashed line represents the graphical estimation of IC_50_.

Since flurbiprofen is a recognized inhibitor of COX-1 and COX-2 [24], we investigated whether Alda-1 exhibits a similar inhibitory effect on these enzymes. We tested concentrations ranging from 0.001 to 32 μM for both flurbiprofen and Alda-1. The results showed that flurbiprofen is a potent inhibitor of COX-1 activity, whereas Alda-1 does not inhibit COX-1 significantly (Figure 5A). The IC_50_ value of flurbiprofen for COX-1, calculated from the obtained data, was 0.017 μM; for Alda-1, it could not be determined. Similar results were obtained for the effects of the compounds on COX-2 activity. The results showed that flurbiprofen inhibited COX-2 with an IC_50_ of 0.19 μM, whereas Alda-1 showed no significant inhibition of COX-2 at any concentration tested (Figure 5B).

**Figure 6:**
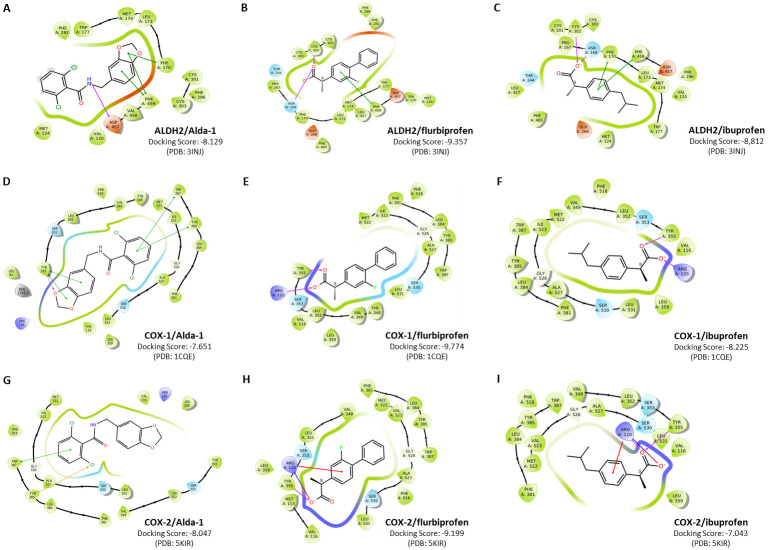
**Molecular docking analysis of Alda-1, flurbiprofen, and ibuprofen interactions with ALDH2, COX-1, and COX-2.** (**A**) ALDH2/Alda-1 complex showing π-π interactions with Phe170 and Phe459, and hydrogen bonding with Asp457 (docking score: −8.129 kcal/mol). (**B**) ALDH2/flurbiprofen complex showing hydrogen bonds with Asn109 and Cys302, and π-π interactions with Phe459 (docking score: −9.357 kcal/mol). (**C**) ALDH2/ibuprofen complex showing hydrogen bonds with Asn109 and Cys302, and π-π interactions with Phe170 and Phe459 (docking score: −8.812 kcal/mol). (**D**) COX-1/Alda-1 complex showing hydrogen and π-π bonds with Tyr355, and π-π interactions with Trp387 and Tyr385 (docking score: −7.651 kcal/mol). (**E**) COX-1/flurbiprofen complex showing hydrogen bonds with Arg120 and Tyr355 (docking score: −9.774 kcal/mol). (**F**) COX-1/ibuprofen complex showing hydrogen bonds with Arg120 and Tyr355 (docking score: −8.225 kcal/mol) (**G**) COX-2/Alda-1 complex showing π-π interactions with Tyr385 and Trp387 (docking score: −8.316 kcal/mol). (**H**) COX-2/flurbiprofen complex showing hydrogen bonds with Arg120 and Tyr355 (docking score: −9.199 kcal/mol). (**I**) COX-2/ibuprofen complex showing hydrogen bonds with Arg120 and Tyr355 (docking score: −7.043 kcal/mol). Key amino acid residues involved in ligand binding are highlighted, with hydrogen bonds (red arrows) and π-π stacking (green endpoint line segment) interactions indicated. S-flurbiprofen and S-ibuprofen were used as the ligand in all docking simulations.

To further investigate the molecular interactions of Alda-1 and flurbiprofen, we conducted molecular docking simulations with the enzymes ALDH2, COX-1, and COX-2. It is important to note that docking scores represent the predicted Gibbs free energy of binding (ΔG), providing a theoretical estimate of affinity; thus, more negative values indicate a thermodynamically more stable complex. For ALDH2 interactions, the analysis revealed that S-flurbiprofen exhibits a highly favorable docking score (−9.774 kcal/mol), higher than that of Alda-1 (−8.129 kcal/mol). Structurally, flurbiprofen is anchored by hydrogen bonds with Cys302 and Asn169, while the fluorophenyl ring establishes stabilizing pi-interactions with Phe459 (Figure 6B). Ibuprofen also binds to Cys302/Asn169 and forms pi-interactions with Phe170 and Phe459, resulting in lower docking scores (−8.812 kcal/mol) (Figure 6C). This difference (~1.0 kcal/mol) suggests that the single isobutyl-phenyl ring of ibuprofen provides substantially weaker hydrophobic stabilization than the biphenyl structure of flurbiprofen. For COX-1 and COX-2 interactions, flurbiprofen displayed the canonical binding mode in both isoforms, forming critical interactions with the gating residues Arg120 and Tyr355, with consistently high scores (−9.774 kcal/mol for COX-1; −9.199 kcal/mol for COX-2) (Figure 6E,H). In contrast, ibuprofen exhibited significantly lower binding energies, particularly for COX-2 (−7.043 kcal/mol). Notably, Alda-1 failed to establish the critical anchor interaction with Arg120 in COX enzymes, explaining its lack of inhibitory activity (Figure 6D,G).

## 3. Discussion

The results of this study show that Alda-1 and flurbiprofen increase ALDH2 activity in PC12 cells, with similar potency at equivalent concentrations. The other NSAID studied, ibuprofen, did not increase ALDH2 activity in PC12 cells. The in vivo results obtained in UChB rats are consistent with those observed in PC12 cells, as Alda-1 and flurbiprofen similarly increased ALDH2 activity in the brain (~4-fold) and in hepatic tissue (~3-fold). In contrast, ibuprofen showed only a slight increase (1.6-fold) in ALDH2 activity in the brain. These effects translated into a decrease in alcohol consumption both in the acquisition phase and in the chronic consumption phase in UChB rats treated with Alda-1 and flurbiprofen. Ibuprofen decreased alcohol consumption during the chronic phase, despite not significantly increasing ALDH2 activity in vitro and in vivo. In search of a common anti-inflammatory mechanism, we found that Alda-1 did not affect COX-1 or COX-2 activity. These findings were supported by in silico docking studies showing fewer interactions between Alda-1 and the active sites of COX-1 and COX-2 than those observed with flurbiprofen.

In vitro assays showed that exposing PC12 cells to 40 μM Alda-1 increased ALDH2 activity by 2.6-fold, consistent with a recent report showing a 1.75-fold increase in ALDH2 activity in HEPG2 cells exposed to the same concentration of Alda-1 [25]. In vitro, we observed that 40 μM flurbiprofen increased ALDH2 activity in PC12 cells by 2.2-fold, consistent with Hosoi et al. [20], who reported a significant 5-fold increase in ALDH2 activity in HEK293 cells after incubation with 100 μM flurbiprofen. We expect that higher concentrations of flurbiprofen will produce greater ALDH2 activation in PC12 cells. Additionally, exposing PC12 cells to varying concentrations of ibuprofen did not alter ALDH2 activity, suggesting that structural differences between flurbiprofen and ibuprofen may explain their differing effects on ALDH2.

Previous studies have demonstrated that activation of ALDH2 via Alda-1 administration markedly reduces both the acquisition and maintenance of chronic ethanol intake in female UChB rats [18]. Furthermore, Alda-1 is highly effective in inhibiting relapse-like ethanol consumption, almost completely abolishing intake following a period of alcohol deprivation [19]. Importantly, these inhibitory effects appear to be specific to ethanol, as Alda-1 administration does not alter voluntary saccharin consumption [18]. Given the previous antecedents, we examined whether flurbiprofen could reduce alcohol consumption in UChB drinking rats. We observed that all tested flurbiprofen doses (2.5, 5, and 10 mg/kg) reduced voluntary alcohol intake by approximately 65%. This protective effect of flurbiprofen was not dose-dependent, indicating that maximal efficacy was achieved at the lowest dose tested and suggesting that flurbiprofen is highly potent in reducing alcohol intake. The flurbiprofen doses used were within the ranges reported in previous studies assessing its efficacy and pharmacokinetics in rats using the same oral route [26,27].

After establishing the effect of flurbiprofen on alcohol intake acquisition, we evaluated the effects of flurbiprofen, along with Alda-1 and ibuprofen, on alcohol consumption after 30 days of chronic intake. We found that oral administration of 5 mg/kg/day of flurbiprofen or Alda-1 reduced alcohol intake by ~60%, an effect that persisted for approximately 5 days after treatment discontinuation. If the prolonged effect of the drugs is due to a long half-life of the compounds or to some taste aversion that developed during repeated oral gavage, this should be determined. Although the ability of Alda-1 to reduce alcohol intake is documented in UChB rats [18,19], this is the first report demonstrating that flurbiprofen can also exert protective effects against alcohol intake. Interestingly, ibuprofen also reduced chronic alcohol intake but to a lesser extent (~25%) compared to Alda-1 and flurbiprofen. The analysis of the effect of treatment on ALDH2 tissue activity showed that both Alda-1 and flurbiprofen markedly increased ALDH2 activity in the liver and brain. In contrast, ibuprofen had a minor effect only on brain ALDH2 activity. These results are consistent with previous studies demonstrating that flurbiprofen and ibuprofen can cross the blood–brain barrier and reach the brain [28]. Although no pharmacokinetic studies have shown that Alda-1 crosses the blood–brain barrier, its high lipophilicity (logP = 4) and prior reports of activating brain ALDH2 [18,29] support the assumption that it can reach the central nervous system. We also identified an inverse correlation between brain ALDH2 activity and alcohol consumption in the animal model used in the present study, consistent with previous reports implicating increased elimination of acetaldehyde, the primary metabolite of alcohol, in reduced rewarding effects of alcohol [30]. The mechanism by which acetaldehyde mediates the pleasurable effects of alcohol is currently unknown. However, studies suggest that this action may be mediated by salsolinol, a condensation product of acetaldehyde and dopamine that acts as an μ-opioid receptor agonist in vitro [31]. Further studies are needed to determine whether Alda-1 or flurbiprofen reduces brain levels of alcohol-derived acetaldehyde or its dopamine condensation product, salsolinol.

The observation that ibuprofen partially decreases alcohol consumption in rats, despite lacking ALDH2-activating properties, suggests that anti-inflammatory mechanisms may contribute to reducing alcohol preference in rats. Therefore, similar pathways could be involved in the marked protective effects against alcohol consumption observed with flurbiprofen. Supporting this assumption, a prior investigation in alcohol-preferring UChB rats demonstrated that aspirin, another NSAID, decreased alcohol intake by roughly 50% [32]. Animal studies have attributed the protective actions of NSAIDs against alcohol consumption to their ability to mitigate the oxidative stress and neuroinflammation associated with alcohol intake [33], a pathological state that has been proposed to play a critical role in the progression of chronic alcohol consumption and relapse [34,35]. The mechanisms by which NSAIDs counteract these neurotoxic effects of alcohol are not clearly known. Still, we speculate that they stem from the inhibition of the metabolism of the high levels of dopamine induced by alcohol intake by COX-1/2, since this catabolic process is associated with the generation of strong oxidizing species such as superoxide and hydrogen peroxide [36]. The different efficacy between flurbiprofen and ibuprofen in decreasing alcohol consumption may not only be associated with their different capacity to activate ALDH2 but also with their different potency to inhibit COX1/COX2: 0.05–0.5 μM for flurbiprofen [24,37] and 7–20 μM for ibuprofen [24]. Since both compounds were administered at the same dose (5 mg/kg), they may have reached brain concentrations that favor COX-1/2 inhibition relative to flurbiprofen.

Considering the apparent significant role of COX-1/2 inhibition in the protective actions of flurbiprofen and ibuprofen against alcohol consumption, we tested whether Alda-1 could also display this pharmacological effect on COX-1/2. We found that Alda-1 does not inhibit COX-1 or COX-2 across concentrations ranging from 0.001 to 30 μM. When flurbiprofen was tested, it showed apparent inhibitory effects on COX-1 and COX-2, with IC_50_ values of 0.017 and 0.19 μM, respectively, consistent with previous reports [24,37]. These results suggest that, in the present study, ALDH2 activation is likely a major contributor to the reduction in alcohol intake, but additional anti-inflammatory or neuromodulatory mechanisms cannot be excluded. Further experiments with COX-2-selective inhibitors could shed light on the relative contributions of both COXs to the inhibition of alcohol intake in rats. In fact, the administration of the selective COX-2 inhibitor celecoxib was shown to reduce the alcohol-induced memory impairment generated in Wistar rats by chronic exposure to alcohol intake [38].

Docking simulations were used to model potential binding modes of Alda-1 and flurbiprofen to ALDH2 and COX enzymes. In ALDH2, the simulations predict that both compounds share hydrophobic interactions with Phe459, a residue previously identified as essential for substrate positioning [39,40]. Flurbiprofen was also observed in silico to interact with Cys302, suggesting a putative mechanism for protecting the catalytic site from 4-HNE-mediated inactivation [41]. Conversely, the docking scores for COX-1/2 favored flurbiprofen, which provides a computational rationale for the observed lack of inhibition by Alda-1. While both compounds are predicted to interact with Tyr355, our model suggests that Alda-1 fails to form the critical hydrogen bond with Arg120 typically required for the potency of carboxylic acid-containing NSAIDs [42]. Furthermore, the interaction of Alda-1 with Tyr355 appears limited to a low-energy π-π interaction. These docking-derived insights serve as a hypothesis-generating framework. While they offer a plausible explanation for why Alda-1 does not disrupt COX activity, compared with the high-energy hydrogen-bonding predicted for flurbiprofen [43], these functional roles require future biochemical validation.

Flurbiprofen is a racemic mixture composed of R- and S-flurbiprofen. However, the two enantiomers exhibit distinct pharmacological properties. The majority of the anti-inflammatory effect is associated with S-flurbiprofen, whereas R-flurbiprofen exhibits weak inhibition of COX-1/2 [44]. In the present study, in vitro and in vivo experiments were performed using flurbiprofen as the racemic mixture (50:50), whereas molecular docking simulations used S-flurbiprofen as the ligand for ALDH2 and COX-1/2. We focused in silico analysis on S-flurbiprofen, the pharmacologically active enantiomer for COX inhibition, thereby enabling a direct structural comparison between the inflammatory and ALDH2-mediated mechanisms. However, since the racemic mixture was effective in vivo, the potential contribution of R-flurbiprofen to ALDH2 activation cannot be ruled out, and enantioselective studies warrant future investigation. Regarding the S-flurbiprofen enantiomer, recent studies have shown that it can also exert antidepressant effects in rats by reducing negative feedback in serotonergic neurons and increasing synaptic serotonin levels [45,46]. Indeed, serotonin reuptake inhibitors that also increase extracellular levels of serotonin, such as fluoxetine, have been associated with a reduction in drinking in patients with alcohol use disorders [47,48]. Considering these antecedents, future preclinical studies are warranted to assess the potential protective effects of S-flurbiprofen against the rewarding and reinforcing effects of alcohol in animal models.

Some limitations of the present study should be noted. First, the effects of the drugs were tested in UChB rats, a rat strain selectively bred for their high alcohol drinking preference. Since UChB rats are highly genetically homogeneous, future studies should test whether these protective effects are replicated in other outbred rodent strains. Second, to confirm the activation of brain ALDH2 as the main mechanism of the drugs for reducing alcohol intake, controls including concurrent central knockout or pharmacological inhibition of ALDH2 would be needed. Third, the relatively small sample size of animal alcohol intake may limit the statistical power to detect smaller effect sizes. Based on the experimental findings, future studies would use operant self-administration and conditioned place preference (CPP) to confirm whether the reduction in ethanol intake induced by Alda-1 and flurbiprofen is associated with a reduction in the rewarding value of alcohol.

The present study validates our initial hypothesis that flurbiprofen reduces voluntary alcohol intake in rats, with evidence suggesting this protective effect primarily operates through increased ALDH2 activity. Our findings indicate that the anti-inflammatory properties of flurbiprofen play a secondary, less critical role in reducing alcohol consumption. These results provide important support for developing ALDH2 activators as a therapeutic strategy for alcohol use disorders. Notably, this study represents the first demonstration of the protective effects of flurbiprofen against alcohol intake. Given that flurbiprofen is an FDA-approved medication with well-established safety and tolerability profiles, repurposing it for this indication would offer significant advantages over developing novel experimental compounds, including reduced development time, lower costs, and an existing evidence base for clinical translation.

## 4. Materials and Methods

### 4.1. Animals

Twenty male Wistar-derived alcohol-preferring UChB rats of approximately 16 weeks’ age and 180–200 g at the start of the experiments, sourced from the vivarium of the Faculty of Medicine, Universidad de Chile, were used in the study. UChB rats fulfill the key criteria for an animal model of alcohol use disorders (AUD) [49] and have previously been shown to be responsive to drugs for the treatment of AUD [50,51]. Rats were individually housed in polycarbonate cages in temperature- and humidity-controlled rooms, under a standard 12 h light–dark cycle (lights off at 7:00 PM), with food and water available ad libitum. To determine the minimum sample size required for the study, we used the following formula for continuous variables: *n* = 1 + 2 × C × (s/d)^2^ [52]. By setting a statistical significance (α) of 0.05 and a power (β) of 0.9, the constant C was established at 10.51. Using variance (s = 1.5 g/kg/day) and a target difference (d = 3.5 g/kg/day) derived from previous data [18], the calculation concluded that a minimum of 5 rats per group is necessary to detect a 50% reduction in alcohol consumption.

### 4.2. Drugs

Alcohol solutions for drinking studies were prepared daily using tap water. Flurbiprofen and ibuprofen were obtained from Sigma-Aldrich (St. Louis, MO, USA), and Alda-1 was synthesized in-house as described previously [18]. All compounds were dissolved in DMSO for in vitro experiments and suspended in a 2% aqueous solution of Arabic gum for intragastric administration to animals.

### 4.3. Exposure of PC-12 Cell Cultures to Drugs

Rat pheochromocytoma PC-12 cells (ATCC CRL-1721) were used to assess in vitro the effects of drugs on ALDH2 activity. PC-12 cells were grown in Roswell Park Memorial Institute (RPMI) medium supplemented with 2 mg/mL NaHCO_3_, 100 U/mL penicillin, 0.1 mg/mL streptomycin, 10% fetal bovine serum, and 5% equine serum. Cells were seeded into 3.5 cm-diameter polystyrene plates and incubated at 37 °C in a 5% CO_2_ atmosphere. When cells reached at least 80% confluence, the culture medium was replaced with medium containing the drugs at 10, 20, or 40 μM or the vehicle (DMSO) at 0.5%, and the cells were incubated for 2 h. Subsequently, the medium was aspirated, and the cells were detached with trypsin. Cells were pelletized by centrifugation at 1000× *g* for 5 min. The supernatant was discarded, and the cells homogenized by pipetting in 300 μL of lysis buffer (100 mM TrisHCl, pH 8.0; 10 mM DTT; 20% glycerol, and 1% Triton X100) [53], followed by one cycle of 5 pulses of ultrasound (Omniruptor 250, Omni Int., Kennesaw, GA, USA). The homogenate was centrifuged at 13,000× *g* at 4 °C for 20 min, and the supernatant was collected for ALDH2 activity determination. The total protein content of the supernatants was determined using a BCA kit (Pierce) according to the manufacturer’s instructions.

### 4.4. Effect of Flurbiprofen on Alcohol Drinking Acquisition by UChB Rats

**Figure 7:**
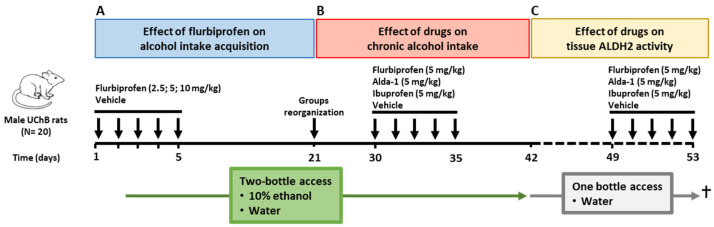
**Experimental timeline and pharmacological treatment protocols.** Twenty male UChB rats were distributed into four groups (*n* = 5/group). (**A**) Alcohol acquisition phase (Days 1–21): Effect of flurbiprofen (2.5, 5, and 10 mg/kg) and vehicle on the initiation of alcohol intake using a two-bottle choice paradigm (10% ethanol vs. water). On day 21, groups were reorganized. (**B**) Chronic alcohol phase (Days 30–42): Assessment of flurbiprofen (5 mg/kg), Alda-1 (5 mg/kg), ibuprofen (5 mg/kg), and vehicle effect on established chronic alcohol intake. (**C**) Tissue Analysis (Days 49–53): Following a period of one-bottle access (water), subjects were treated with the drugs (flurbiprofen, Alda-1, ibuprofen, and vehicle) and euthanized to determine their effect on brain and liver ALDH2 activity. The black cross indicates the day of animal euthanasia.

Rats were assigned to 4 groups (*n* = 5) to receive daily intragastric (i.g.) doses of 2.5, 5, or 10 mg of flurbiprofen or vehicle (2% Arabic gum) in approximately 1.5 mL for 5 consecutive days. On the second day of treatment, alcohol access began under a 24-h, two-bottle, free-choice paradigm of water and 10% alcohol, maintained until day 20. Daily fluid intake was recorded, and the alcohol intake was expressed as g alcohol/kg/day (Figure 7).

### 4.5. Effect of Flurbiprofen, Alda-1, and Ibuprofen on Chronic Alcohol Intake of UChB Rats

On day 21 of the experiment, animals were randomly reorganized into four new groups and continued the free-choice drinking paradigm until day 46. Beginning on day 30, after 9 days of stable baseline alcohol consumption, animals were administered (i.g.) daily doses of 5 mg/kg of flurbiprofen (*n* = 5), Alda-1 (*n* = 5), ibuprofen (*n* = 5), or vehicle (*n* = 5) for 5 days. Daily fluid intake was recorded, and the alcohol intake was expressed as g ethanol/kg/day. Seven days after finishing alcohol drinking, animals were exposed to a new cycle of drug administration. One hour after the last dose, animals were euthanized, and the brain and liver were excised. Approximately 0.5 g of tissue was cut into small pieces, washed with PBS at 0 °C, and homogenized by sonication (four cycles of 5 pulses) in 5 volumes of lysis buffer (1% Triton X-100, 2 mM DTT). The homogenate was centrifuged at 20,800× *g* at 4 °C for 20 min, and the supernatant was collected to determine total protein content and ALDH2 activity (Figure 7).

### 4.6. ALDH2 Activity Measurement

ALDH2 activity was measured as previously described by Rivera-Meza et al. [18]. Briefly, enzymatic activity was estimated in duplicate by measuring the generation of NADH at 340 nm at 35 °C in a mix containing a volume of homogenate equivalent to approximately 200 μg of protein, 34 mM Na_2_HPO_4_ (pH 8.5), 10 mM Pirazol, 5 mM MgCl_2_, 4 mM DTT, 0.8 mM NAD^+^, and 21 μM propionaldehyde. Enzymatic activity was expressed as nmol NADH/min/mg protein.

### 4.7. Effect of Alda-1 and Flurbiprofen on COX-1 and COX-2 Activity

A COX-1/2 Colorimetric Inhibitor Screening Assay Kit (Cayman Item No. 701050) was used to evaluate the inhibitory activity of Alda-1 and flurbiprofen. For the evaluation of COX-1 and COX-2 inhibitory activity, each sample was prepared in triplicate at a concentration of 0.001, 0.0032, 0.01, 0.032, 0.1, 0.32, 1, 3.2, 10, 32, and 100 μM. The assay was started with a 5 min incubation at 25 °C. After incubation, 100 μM arachidonic acid was added to all wells to initiate the reaction. Peroxidase activity was monitored by measuring absorbance at 590 nm for 2 min using an Epoch (Biotek Instruments, Winooski, VT, USA) microplate reader. Results were expressed as IC_50_ values, defined as the concentration at which 50% inhibition of enzyme activity occurred for COX-1 and COX-2. The IC_50_ values were calculated according to the manufacturer’s instructions.

### 4.8. In Silico Analysis

The crystallographic structures of human ALDH2 (PDB ID: 3INJ), ovine COX-1 (PDB ID: 1CQE; used as a structural surrogate for the human enzyme), and human COX-2 (PDB ID: 5KIR) were retrieved from the RCSB Protein Data Bank. Protein preparation was performed using AutoDock Tools (MGLTools v.1.5.6); water molecules and co-crystallized ligands were removed, polar hydrogen atoms were added, and Kollman united atom charges were assigned. The 3D structures of Alda-1, S-flurbiprofen, and S-ibuprofen were constructed, and their geometries were optimized using the MMFF94 force field. Molecular docking simulations were executed using AutoDock Vina v.1.2.5. Grid boxes were defined with dimensions of 25 × 25 × 25 Å, centered on the coordinates of the original co-crystallized ligands. The binding affinity (kcal/mol) was used to rank the poses. Post-docking analysis and the generation of 2D and 3D interaction diagrams to identify key hydrogen bonds and pi-pi stacking interactions were performed using BIOVIA Discovery Studio Visualizer 2026.

### 4.9. Statistical Analysis

The data are presented as the means ± SEM. The normality of the data was confirmed using the Shapiro–Wilk Normality Test. ALDH2 activity in PC12 cells and rat tissues was analyzed using a one-way ANOVA, followed by Dunnett’s and Tukey’s post hoc multiple comparison tests, respectively. Correlation between brain ALDH2 activity and alcohol consumption was determined by linear regression analysis. Alcohol and total fluid consumption data were analyzed using repeated-measures (RM) ANOVA, with treatment as the between-subjects factor and alcohol solution or total fluid consumption as the dependent variable, followed by a Bonferroni post hoc test. Inhibitory concentration 50 (IC_50_) of Alda-1 or flurbiprofen on COX-1/2 was determined by nonlinear regression. All statistical analyses were performed using GraphPad Prism 8 software (GraphPad Software Inc., San Diego, CA, USA). A *p*-value of less than 0.05 was considered statistically significant.

## Data Availability

The original data presented in the study are openly available in https://doi.org/10.34691/UCHILE/1CURKB.
